# Human exposure to nitro musks and the evaluation of their potential toxicity: an overview

**DOI:** 10.1186/1476-069X-13-14

**Published:** 2014-03-11

**Authors:** Kathryn M Taylor, Marc Weisskopf, James Shine

**Affiliations:** 1 Department of Environmental Health, Harvard School of Public Health, 677 Huntington Avenue, Boston, MA 02115, USA; 2 Department of Epidemiology, Harvard School of Public Health, 677 Huntington Avenue, Boston, MA 02115, USA

**Keywords:** Nitro musks, Fragrance, Persistent pollutant, Review

## Abstract

Synthetic nitro musks are fragrant chemicals found in household and personal care products. The use of these products leads to direct exposures via dermal absorption, as well as inhalation of contaminated dust and volatilized fragrances. Evidence also suggests that humans are exposed to low doses of these chemicals through oral absorption of contaminated liquids and foods. As these compounds are lipophilic, they and their metabolites, have been found not only in blood, but also breast milk and adipose tissue. After personal use, these environmentally persistent pollutants then pass through sewage treatment plants through their effluent into the environment.

Little is known about the biological effects in humans after such a prolonged low dose exposure to these chemicals. While epidemiologic studies evaluating the effects of nitro musk exposures are lacking, there is limited evidence that suggest blood levels of nitro musks are inversely related to luteal hormone levels. This is supported by animal models and laboratory studies that have shown that nitro musks are weakly estrogenic. Nitro musks exposure has been associated with an increased risk of tumor formation in mice. The evidence suggests that while nitro musks by themselves are not genotoxic, they may increase the genotoxicity of other chemicals. However, animal models for nitro musk exposure have proven to be problematic since certain outcomes are species specific. This may explain why evidence for developmental effects in animals is conflicting and inconclusive. Given that animal models and cell-line experiments are suggestive of adverse outcomes, further epidemiologic studies are warranted.

## Introduction

**Figure 1:**
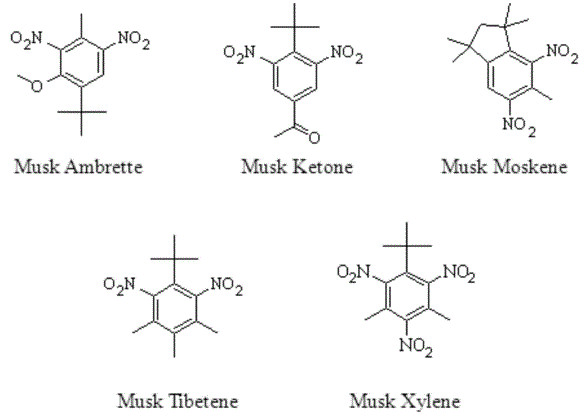
Structural formulas of the five most commercially relevant synthetic nitro musks.

Synthetic nitro musks are alkylated nitrobenzene derivatives. These chemicals are an anthropogenic component in fragrant compositions. They were synthesized in the early 1900s as inexpensive substitutes for natural macrocyclic musks used in perfumes [1]. Nitro musks generally refer to the five most commercially relevant fragrant compounds (Figure 1): ketone musk (4-tert-butyl-2,6-dimethyl-3,5-dinitroacetophenone), musk ambrette (2,6-dinitro-3-methoxy-4-tert-butyltoluene), musk moskene (1,1,3,3,5-pentamethyl-4,6-dinitro-2H-indene), musk tibetene (1-tert-butyl-3,4,5-trimethyl-2,6-dinitrobenzene) and musk xylene (1-tert-butyl-,5-dimethyl-2,4,6-trinitrobenzene). Musk moskene and musk tibetene have been prohibited from use in fragrant products because of adverse outcomes from structurally similar compounds [2]. Musk ambrette has been discontinued from use because its consumption was associated with hind limb weakness in rats and observed neuropathologic changes in the brain, spinal cord and peripheral nerves [3]. Ketone musk and musk xylene continue to be used as additives in detergents, fabric softener, household cleaning products and other fragrant non-cosmetic products with musk xylene being the most widely used nitro musk [4].

In 2008, under the authority of the Registration, Evaluation, Authorization and Restriction of Chemicals (REACH) regulation, musk xylene was classified as a substance of high concern with a very persistent, very bioaccumulative (vPvB) designation. A restricted use warning was placed on musk ketone. They found that nitro musk compounds do not degrade easily, causing them to be highly stable and ubiquitous in the environment [5].

Nitro musks have been largely replaced by polycyclic musks due to banning of these compounds in several countries [6,7]. However, nitro musks are still being produced in China and India and used in non-cosmetic compounds in the United States that have not been reformulated. Given the environmental persistence and the continued use of nitro musks even at a decreased level, there are concerns for the effects of long-term exposure. This overview is designed to evaluate the literature on nitro musks and discuss areas for further research.

## Background

### Human exposures

The primary source of exposure to synthetic musks is through exposure to scented personal care products or scented household goods. In a report evaluating the concentrations of synthetic musks in personal care products in China, Lu et al. found musk xylene in 19% of the products and they found musk ketone in 57% of the products tested. These products included hair care products, body washes, toilet soaps, skin lotions and make up. The highest concentration of musk xylene was found in skin lotions with a mean concentration of 0.16 ug/g of lotion. The highest concentration of musk ketone was found in hair care products with a mean concentration of 8.12 ug/g of product [8].

The three main routes of exposure to nitro musks are through inhalation, dermal absorption and ingestion. Lu et al. [9] tested dust from a random sampling of homes in China finding musk ketone in 98.7% of the homes at a median concentration of 13.7 ng/g and musk xylene at a median concentration of 11.8 ng/g in 86.4% of the homes. This raises concern for inhalation of nitro musks through residual dust in the home [9]. However, human exposure to nitro musks via inhalation from the environment has been found to be minimal suggesting that inhalation from direct use is probably a more important exposure [10].

Dermal exposure to fragrances was thought to be an important route of exposure to musk. However, evidence suggests that absorption of dermally applied nitro musks is low. In a study by Hawkins et al. [11] when musk ketone and musk xylene were separately applied dermally to the backs of seven participants, the study found that after 6 hours, they were able to recover 86% of applied musk ketone with only 0.49% found in the urine and less than 0.01% in the feces and they were able to recover a range of 90-94% of musk xylene with 0.2-0.3% excreted in the urine and none found in the feces. Neither chemical was found in the blood. Hutter et al. [12] reached similar findings when they evaluated the association between dermal absorption through cosmetic use and blood levels of synthetic musks. Lignell et al. [13] looked at the effect of dermal absorption of nitro musks through perfume application in lactating women. They found that perfume use during pregnancy was not predictive of levels of musk ketone and musk xylene in breast milk. However, Eisenhardt et al. [14] found that blood levels of nitro musk were associated with use of cosmetics, in particular with perfume use, which would suggest an association with dermal absorption but also could be due to inhalation. However, it appears they did not control for strong predictors of lipophilic chemical concentrations in the blood such as age, number of children breastfed or Body Mass Index when evaluating this relationship. This suggests that percutaneous absorption might not be as significant as initially thought, and so should be controlled for in exposure analyses but might not be as important of a route of exposure.

Considering that most nitro musks have been replaced by polycyclic musks in many products applied dermally, oral exposure through contaminated water or food might be a big contributor to nitro musk presence in the blood, breast milk, and lymphocytes [4]. This is supported by a study performed by Riedel and Dekant [15] in which both dermal and oral exposures to musk xylene were assessed in 12 volunteers over a 96 hour exposure. The percentage of dose administered found in plasma was an order of magnitude greater for oral exposure when compared with dermal exposure. Nitro musks have been found at concentrations ranging from below the limit of detection to 470 ng/g of lipid weight in freshwater fish samples, a result of sewage runoff into aquatic systems. Because of this consumption of contaminated fish may be an important exposure to consider [16]. However, while varying levels of nitro musks have been found in fish samples, Kafferlein and Angerer found that dietary intake of fish, assessed by a food frequency questionnaire, was not correlated with blood levels of nitro musks [1,17,18].

Most human studies involving nitro musks have looked to assess the toxicokinetics of the chemicals once the nitro musks are absorbed. Upon absorption by the body, musk xylene can go through a reduction process that produces nitro derivatives. One of these derivatives, 1-tert-butyl-3,5-dimethyl-4-amino-2,6-dinitrobenzene has been shown to bind to hemoglobin and is a biotransformation product that is often found in urine [19]. The half-life of musk xylene ranges from 60-94 in the body [20]. This long half-life can be explained by a two compartment kinetic model where musk is either initially biotransformed by the body and excreted or distributed by the blood into a second compartment, in this case, the adipose tissue [15].

Nitro musks have a low range (4.3-4.9) of octanol-water partition coefficients indicating that they are lipophilic [21]. In 1994, studies began finding the presence of nitro musks in human adipose tissue and milk samples. Levels of nitro musks ranged from 0.01 to 0.22 mg/kg of fat in adipose samples and 0.01-0.19 mg/kg of fat in milk samples [22]. In a study performed in Massachusetts on breast milk, Reiner et al. [23] found similar results with ranges of nitro musks found between 0.02-0.238 mg/kg of fat. Maternal age was not correlated with the levels of musks in breast milk, which is not consistent with most persistent organic pollutants. There was an inverse relationship between number of children breastfed previously and breast milk concentration, which is to be expected with lipophilic pollutants. However, this relationship was not significant.

### Musks in the environment

Although human use of household and personal care products containing nitro musks is the primary route of human exposure, it was the presence of these compounds in environmental samples that originally drove concern about the biological and environmental effects of nitro musks in the 1980s. Yamagishi et al. [17] found their presence in more than 80% of freshwater fish samples, river water, and waste water taken from multiple sampling stations on the Tama River and Tokyo Bay. Since then nitro musks have been found in the biota and marine environments of the Baltic Sea, the North Sea, the Vlatava River in Prague, and Lake Michigan in the U.S. [1,24,25].

Nitro musks are not easily degradable, which accounts in part for their widespread presence in environmental samples. Nonetheless when nitro musks make their way from personal use settings and into wastewater, passage through sewage treatment plants does appear to degrade the nitro musks somewhat as by-products of nitro musks have been found in sampled sewage treatment plant effluents [6]. However, sewage treatment plants only degrade between 46-54% of the nitro musks that enter the plant prior to releasing their effluent back into the environment [7]. Therefore, even with such treatment, the potential for environmental exposure remains. Furthermore, the biotransformation products of nitro musks created by the sewage treatment process might also be of interest in risk assessment. Biotransformation products of nitro musks have been found in aquatic systems at higher concentrations than the parent compound indicating that transformed products of nitro musks are also environmentally persistent [17,24,26].

Wastewater effluent has the ability to affect the concentrations of chemicals in surface water. This is of importance when contaminated water sources are used to supply water treatment plants or where they are used to recharge groundwater. Herberer et al. [27] found that some organic pollutants found in tap water were correlated with the levels of surface water filtration and intentional ground water replenishment in Berlin. In the United States, recycled wastewater is currently used for indirect potable reuse and non-potable reuse, meaning reused water is not directly used for tap water. However, a report put out by the National Committee on the Assessment of Water Reuse as an Approach to Meeting Future Water Supply Needs [28] declared that reused water could be used to augment surface water supplies which could be used for drinking water sources. This would create a closed water system loop or a semi-closed water system loop. This raises concern over how this will affect the concentrations of nitro musks and other environmentally stable pollutants in drinking water. Evidence suggests that areas that use closed or semi-closed looped water replenishment systems see a gradual increase of contaminant concentration over time. This effect is a result of low surface water flow and increased sewage effluent being added to surface waters [27,29].

An interesting secondary source of nitro musks to the environment, specifically musk ketone, may be melting glaciers. This was demonstrated in Lake Oberaar, a glacier fed lake where spikes of persistent organic pollutants were detected in glacier runoff [30]. This was even seen for contaminants whose use had long been discontinued. This suggests that even if nitro musk production was discontinued there is still a potential for exposure.

The persistent nature of musks in the environment has been well documented. Furthermore, a vast number of people may be exposed to these chemicals. Although a consensus has not been reached over the biological effects of the continuous, low-dose exposures humans may experience, it is important that possible health outcomes from such exposures to nitro musks are assessed.

### Toxicity

Animal and laboratory studies of nitro musks have largely focused on three main areas: developmental effects due to perinatal and early childhood exposure, endocrine effects, and carcinogenic effects. A summary of the studies used in this overview can be found in Additional file 1.

Studies of developmental effects have focused on adverse birth outcomes across species, such as the ability to conceive and the viability of the embryos, and have found conflicting results. Exposure of pregnant rats to 45 mg/kg/day of musk ketone or 200 mg/kg/day of musk xylene, was not shown to have any adverse effect on the embryo [31]. In zebrafish, musk ketone between the levels of 0.1 and 10 mg/g of food/day was found to reduce the body weight of spawning females up to 38% and reduce the number of eggs/female/day up to 95% [32]. Embryos that were exposed to musk ketone at levels higher than 10 μg/l in the surrounding water had decreased early life stage survival [32]. When embryos of South African clawed frogs were exposed to 400 μg/l/day of either musk ketone, musk xylene or musk moskene in the surrounding water for 11 days, there was increasing larvae mortality for each increasing day of exposure [33].

Once the lipophilicity of nitro musks was recognized there was general concern over the possible endocrine modulating effects of these chemicals. This is because many similar aromatic, lipophilic compounds have been shown to have endocrine receptor binding capabilities [34,35]. One of the first studies, investigating potential endocrine effects, looked at the binding capability of musk xylene, musk ketone, musk moskene and their derivatives to the estrogen receptors in rainbow trout and the South African clawed frog. There was no observed binding of musk xylene, musk ketone or musk moskene to the estrogen receptors in both species. However, there was competitive binding of the estrogen receptors by three of the derivatives, 4-NH_2_-musk xylene, 2-NH_2_-musk xylene, and 2-NH_2_-musk ketone, in both species after exposure to the chemicals for 20 hours at concentrations of 10^−6^ to 10^−3^ M in surrounding media, which is well above environmentally relevant levels. The binding affinity of these derivatives were up to 375 times lower than estradiol and up to 150 times lower than bisphenol A [36].

Bitsch et al. [37] conducted an E-screen assay to detect estrogenic activity using human MCF-7 breast cancer cells exposed to 10 mmol of either musk xylene or musk ketone per liter of test substance or 5 mmol of other nitro musk derivatives per liter of surrounding media. Estrogenic activity was confirmed by a significant increase in the proliferation of human breast cancer cells by 29% for musk xylene, by 97% for musk ketone and by 29% for p-amino-musk-xylene (a musk xylene derivative) when compared to the negative control. This increased proliferation was eradicated when the musks were added in the presence of 1 umol/L of tamoxifen, an anti-estrogenic drug, indicating that the musk effects were mediated, at least partly by the estrogen receptor. However, relative proliferation potency for the nitro musks in this assay was approximately 30,000 times lower than estradiol.

Increased concern for the potential carcinogenic effects of nitro musks was raised by a study of Maekawa et al. [38], which found that mice fed with food containing 0.075% or 0.15% musk xylene had up to three times as many tumors upon post-mortem inspection than control mice. Carcinogenicity studies after the Maekawa study focused on examining whether these chemicals were genotoxic. Multiple studies using various tests for genotoxicity in rats, E. coli PQ27 cells, human lymphocytes, and human hepatoma cell lines indicated that nitro musks were not genotoxic [39-41]. These results implied that there were other processes occurring *in vivo* to promote tumor genesis.

One potential pathway to explain this increase in tumor genesis after exposure to nitro musks is that it could be a result of nitro musks interaction with other toxins to increase the potency of known genotoxicants. Using the E. coli genotoxicity assay as an indicator of DNA damage, musk ketone, but not musk xylene, was shown to increase the toxicity of benzo-a-pyrene, 2-aminoanthracene and aflotoxin B1 in rats when exposed to levels higher than 10 mg of musk ketone per day [42]. This was confirmed using a micro nucleus test on musk ketone exposed human derived Hep G2 cells. When the Hep G2 cells were exposed to 5–5000 ng/l of musk ketone and 0.2 ug/ml of benzo-a-pyrene, no biological interaction occurred. However, when the cells where exposed to musk ketone 28 hours before the addition of benzo-a-pyrene, there was a synergistic effect showing an increase in the genotoxic effects of benzo-a-pyrene exposure [43]. The enhanced effect of benzo-a-pyrene after musk ketone exposure may be explained by the ability of musk ketone and musk xylene, injected individually, to induce production of CYP-4501A2, CYP-4501A3, and CYP-450IB2 which was initiated when rats were injected at doses starting at 10 mg of each musk per kg of body weight. These enzymes are produced in response to exposure to xenobiotics in order to reduce or oxidize chemicals in the body. This response is usually beneficial but can sometimes have deleterious effects if the xenobiotic becomes more reactive after its transformation [44,45]. In contrast, Schnell et al. [46] found that both musk xylene and musk ketone inhibit the catalytic processes of CYP4501A in carp, suggesting that nitro musk interaction with CYP450 is likely species-specific [44,45].

Another pathway for the promotion of tumor genesis was proposed by Luckenbach and Epel [47]. They found that musk xylene and musk ketone could inhibit the effectiveness of multidrug efflux transporters in marine mussels. A major consequence of this inhibition is loss of the cell’s ability to remove xenobiotics, including known carcinogens, allowing them to remain in the cell longer. The effects of an exposure to 2 hours of synthetic musks took between 24 and 48 hours to be reversed.

### Health outcomes

There has been one case control study that has evaluated possible health outcomes associated with nitro musk exposure. This study by Eisenhardt et al. [14] analyzed the association between musk ketone and musk xylene blood levels and endocrine and gynecological problems in premenopausal women at an endocrinological outpatient clinic. They found that women presenting with premenstrual syndrome had on average 24 ng musk ketone per liter of blood higher than women who did not present with premenstrual syndrome (p = 0.014). They also found that musk xylene levels were inversely associated with levels of the luteal phase hormones, progesterone and estrogen (p = 0.08). Women who presented as being infertile had 23.5 ng/L higher serum levels of musk xylene than those who had already been pregnant once (p = 0.045). However, given that nitro musks are lipophilic the lower levels of musk xylene in fertile women can possibly be explained by elimination of nitro musk through breast feeding. These findings are suggestive and may indicate that nitro musks could be disruptors of the hypothalamic-ovarian hormone pathway and further research is necessary to evaluate this relationship.

## Conclusion

The use of animal models and laboratory studies is important for the risk assessment process. However, it can be difficult to extrapolate how these studies apply to typical everyday exposures of humans. The exposure concentrations of the nitro musks in the various animal models addressed above ranged from 1 μl/L to 5000 μl/L. Considering nitro musks have been found in aquatic systems at lower concentrations and that humans have significantly more body mass than the animals being studied, the studies may not be applicable to human nitro-musk exposure. At the same time, many of these animal studies involved short duration exposures. Although human exposure is likely at lower doses, it also is likely to be long-term. The animal studies do not address this exposure scenario.

The body of literature supports the conclusion that not only are we being exposed to nitro musks, we are also bioaccumulating them and passing them on to our offspring through breast milk and perinatal exposures. While the animal studies do not address long-term low dose effects, they do indicate that a particular area of focus for health outcomes from nitro musk exposure should be tumor genesis and cancer. While animal studies were conflicting for potential developmental effects, this lack of agreement indicates that more research needs to be done in this field. Human endocrine effects have been seen for nitro musk exposures; this indicates that more studies need to be done in animals and humans at environmentally relevant exposure levels. In light of the evidence, the precautionary principle should be taken into account. This can be done through a reduction in the use and production of products containing nitro musks.

## Abbreviations

REACH: Registration, Evaluation, Authorization and Restriction of Chemicals; vPvB: Very persistent, very bioaccumulative.

## Competing interests

The authors declare that they have no competing interests.

## Authors’ contributions

KMT did a systematic review of the literature and drafted the manuscript. MGW participated in the drafting and coordination of the paper and critically revised the manuscript. JPS conceived of the review study and critically revised the manuscript. All gave approval of the final manuscript.

## Supplementary Material

Additional file 1 Summary of nitromusk toxicity studies performed in animals and human cell lines. Click here for file
