# Supplementary material for: Human exposure to nitro musks and the evaluation of their potential toxicity: an overview
Source: Environ Health. 2014 Mar 11;13:14. doi: 10.1186/1476-069X-13-14 (PMC4007519; doi:10.1186/1476-069X-13-14)
Supplement: Additional file 1 — Summary of nitromusk toxicity studies performed in animals and human cell lines. [file 1476-069X-13-14-S1.pdf]

**Appendix 1:** Summary of nitromusk toxicity studies performed in animals and human cell lines.					
**Study**	**Nitro Musk Tested***	**Animal/ Cell Line Model Used**	**Doses Tested**	**Effect Level**	**Outcome tested**
Api et. al 1995	MK	In vivo mouse micronucleus assay	250, 500 or 1000mg/kg body weight evaluated at 24, 48 and 72 hrs	No significant increase in micronucleated polychromatic erythrocytes at any level	Genotoxicity/ Carcinogenicity
Bitsch et. al 2002	MK, MX and 4X	MCF-7 breast cancer cells	5-10mmol/liter in surrounding media	Observed increased proliferation in MCF cells at 10mmol/L of test substance for:<br>MK: by 96% at 10mmol/L of test substance<br>MX: by 26% at 10mmol/L of test substance<br>4X: by 29% at 5mmol/L of test substance	Competitive binding capability to estrogen receptors
Carlsson et. al 2000	MK	Zebrafish (*Danio rerio*)	0.1-10 mg/g of food per day and 1-1000ug/l of surrounding water	Observed decrease in body weight by ~40% at 10 mg MK/g of food<br><br>Observed decrease in number of eggs/female/ day by 50% at 0.1mg MK/g of food and by 95% at 10 mg MK/g of food<br><br>No observed adverse effects for early life mortality up to 10mg/l of surrounding water	Body weight and fecundity of spawning females and early life stage mortality
Chau and Dietrich 1999	MK, MX, MM	Zebrafish (*Danio rerio*) and South African Clawed Frog (*Xenopus laevis*)	400ug/l of surrounding water for 96 hours or for 11 days	No observed adverse effects for viability up to 96 hours of exposure to MK, MX or MM	Larval Mortality
Chau and Dietrich 2000	MK, MX, MM, 4X, 2X, 2K	Rainbow Trout (*Oncorhynchus mykiss*) and South African Clawed Frog (*Xenopus laevis*)	10^{-6}-10^{-3} mol/liter in surrounding media	The concentration of the nitro musk needed to inhibit [3H]estradiol-17 beta specific binding by 50% of the IC_{50} value for:<br>4X: 30.8uM<br>2X: 12.9uM<br>2K: 70.1uM	Competitive binding capability to estrogen receptors
Christian et al. 1999	MK, MX	Sprague- Dawley Rats	60-2000 mg/kg per day	Developmental NOAEL (No Observerved adverse effect level) set at :<br>MK: > 45 mg/kg per day<br>MX: > 200 mg/kg per day	Developmental Toxicity

Iwata et. al 1992	MX	Male Wistar Rats	50, 100 or 200 mg MX/kg of body weight	Observed 30% increase in CYP-450IA2 production at lowest exposure to MX (50mg/kg of body weight)	CYP-450 production
Kevekordes et. al 1996	MX, MK, MA, MM, MT	Hep G2 human hepatoma cells and human lymphocytes	MX: 0.014-270uM<br>MK: 0.014-272uM<br>MA: 0.015-298uM<br>MM: 0.014-288uM<br>MT: 0.018-360uM	No genotoxicity at any exposure in both cell lines	Genotoxicity/ Carcinogenicity
Luckenbach and Epel 2005	MK, MX	California Mussels	0.01-100µM	Observed inhibition of multidrug transporters at 0.1µM for both MK and MX	Inhibition of Multidrug transporters
Maekawa et. al 1990	MX	B6C3F Mice	0 (control), 0.075 or 0.15% of food concentration	Observed a 68% increase in those with tumors at lowest exposure (0.075% of food intake)	Long-term toxicity /carcinogenicity
Mersch-Sunderman et. al 1996	MX, MK	Sprague-Dawley Rats	10, 20 or 40 mg/ day	Increased potency of benzo-a-pyrene, 2-aminoanthracene and aflotoxin B1 at lowest exposure of MK (10mg/day)<br><br>No observed effect in MX	Co-genotoxicity
Mersch-Sunderman et. al 2001	MK	Hep G2 Human Hepatoma Cells	5-5000ng/ml	Increased potency of benzo-a-pyrene starting at 50 ng of MK/l	Co-genotoxicity
Schnell et al 2009	MK, MX	Carp (*cyprinus carpio*)	0.1-1µM	50% inhibition of Cyp activity for MK: 35µM and MX: 37µM	Interaction effect of musk compositions on CYP-450 production
Stuard et. al 1997	MK	B6C3F Mice	5-500 mg/kg of body weight for 7 consecutive days	Observed dose increase of cyp-450IA2, 450IA3 and 450IB2 starting at 10mg/kg of body weight	CYP-450 production
*Abbreviations- MA: Musk Ambrette, MK: Musk Ketone, MM: Musk Moskene, MT: Musk Tibetene, MX: Musk Xylene, 4X: 4-NH2-Musk Xylene, 2X: 2-NH2-Musk Xylene					
